# Frailty’s influence on older stroke patients: Neurological outcome and mortality after endovascular treatment in stroke: A national German stroke registry analysis

**DOI:** 10.1177/23969873251344202

**Published:** 2025-06-11

**Authors:** Marlena Schnieder, Hannah Metz, Mathias Baehr, Anna Alegiani, Silke Wunderlich, Christian H Nolte, Arno Reich, João Pinho, Christiane Huber, Gabor Petzold, Steffen Tiedt, Christine AF von Arnim, Jan Liman

**Affiliations:** 1 Department of Neurology, University Medical Centre Goettingen, Göttingen, Germany; 2 Department of Geriatrics, University Medical Centre Goettingen, Göttingen, Germany; 3 University Medical Centre Hamburg-Eppendorf, Hamburg, Germany; 4 Clinic and Policlinic for Neurology, Hospital of the Technical University Munich, Munich, Germany; 5 Department of Neurology and Centre for Stroke Research Berlin (CSB), Charité - Universitätsmedizin Berlin, Berlin Institute of Health (BIH), Berlin, Germany; 6 Department of Neurology, RTWH Aachen University, Aachen, Germany; 7 Division of Vascular Neurology, Department of Neurology, University Hospital Bonn, Bonn, Germany; 8 Institute for Stroke and Dementia Research, University Hospital, LMU Munich, Munich, Germany; 9 Department of Neurology, Paracelsus Medical University, Nuremberg, Germany

**Keywords:** Endovascular treatment, frailty, functional outcome

## Abstract

**Introduction:**

Frailty is a clinical syndrome particular in old patients with an increased risk of adverse health-care events. In geriatric stroke patients who received endovascular treatment, monocentric analyses have demonstrated that frailty affects mortality and functional outcome. We aimed to investigate the impact of frailty in a larger multicentric cohort.

**Patients and methods:**

We analyzed the impact of frailty on outcome in patients with stroke who underwent endovascular treatment in seven academic centers contributing to the German Stroke Registry. We calculated the Hospital Frailty Risk Score (HFRS) for all patients aged ⩾ 65 years. Functional outcome was measured by modified Rankin Scale (mRS) 3 months after the stroke event. A regression analysis conducted to assess mortality and functional outcome, adjusted for factors known to influence outcomes.

**Results:**

2468 patients fulfilled the inclusion criteria. Median HFRS was 1.1 (IQR 0–2.95) and 449 (18.2%) patients had HFRS > 5. Low, intermediate and high-frailty risk was present in 2009 (71.7%), 389 (15.8%), and 60 (2.44%) respectively. A favorable neurological outcome (mRS 0–2) was achieved in 31.7%, 20.6%, and 13.8% in the low-, moderate, and high-risk-frailty-groups respectively (*p* < 0.001). Multivariate regression analysis showed a significant associations of HFRS on both mortality (adjusted OR 1.033, 95% CI: 1.004–1.063, *p* = 0.024) and functional outcome (adjusted OR: 0.962, 95% CI: 0.929–0.997; *p* = 0.033) after 3 months. However, there was no significant difference in baseline NHISS scores between frail and non-frail patients (14 (IQR 19–19)) vs 15 (IQR 11–19) vs 15 (IQR 10–19); *p* = 0.295). Besides door-to-groin time (DTN) differed with high frailty-risk patients having the longest DTN times (64 (38–102) vs 67.5 (45–95) vs 80 (54–106); *p* = 0.020).

**Discussion and conclusion:**

We identified frailty as a factor strongly associated with both mortality and functional outcome in ischemic stroke patients undergoing thrombectomy.

## Graphical abstract

**Graphical abstract:**
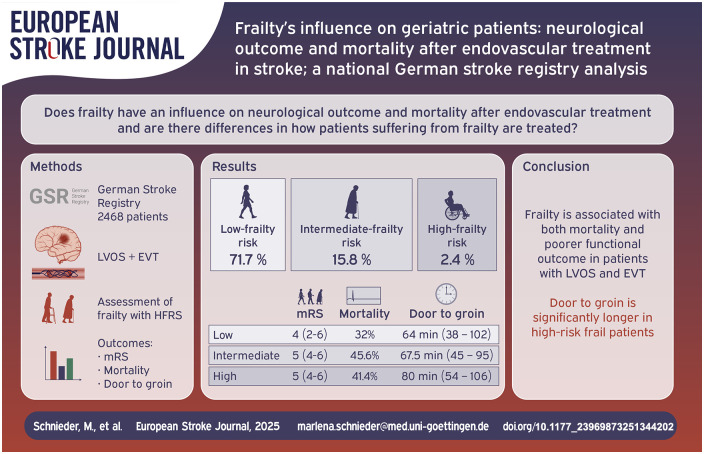

## Introduction

As around 11% of old patients experience frailty, it is essential to understand the impact of frailty in our aging society.^1^ Frailty is even more prevalent among stroke patients, with 24.6% affected.^2^ Frailty is defined as a multidimensional clinical syndrome involving a decline in function in multiple physical systems, resulting in decreased physiological reserve and greater vulnerability to stressors. This makes frailty to a strongly associated with adverse health outcomes and mortality in old patients.^3^ Several factors are linked to frailty: While it is associated with older age, age is not the only factor leading to frailty.^4^ It is more common in women and correlates with lower education and income as well as higher rates of comorbid chronic disease.^5^ However, patients can be frail even without comorbidities, and some individuals can be old and multimorbid without suffering from frailty.^6^ The operational and conceptual definitions of frailty are still being debated, and several methods describing frailty have evolved. The recently adopted Hospital Frailty Risk Score (HFRS) is based on standard diagnostic codes and incorporates routinely collected data.

Stroke is one of the leading causes to long-term disability, and its incidence increases with age.^7^ Significant advances in stroke-care have been made in recent years, including the implementation of mechanical thrombectomy as standard of care for large vessel occlusion stroke (LVOS).^8^ However, mortality rates and the need for long-term care after stroke remain high.^9^ Identifying factors that contribute to poor outcomes is challenging, and several factors as a higher pre-stroke modified Rankin Scale (mRS),^10^ are known to be associated with poor neurological outcomes. Although numerous scores have been proposed to predict outcomes after mechanical thrombectomy, accurately predicting outcome remains difficult. An accurate assessment of frailty could help identify patients at higher risk for a poor outcome and adverse events. Small monocentric analyses have demonstrated frailty’s impact on outcome after stroke treated with mechanical thrombectomy.^11–13^ Our aim was to study the impact of frailty on patients undergoing mechanical thrombectomy for large-vessel occlusion stroke in a large multicenter cohort to investigate whether frailty can function as a predictor of functional outcome and mortality.

## Methods

We conducted a retrospective analysis of data of patients >65 years collected in the German Stroke Registry between 2016 and 2019. The German Stroke Registry-Endovascular Treatment (GSR-ET 07/2015–04/2018; ClinicalTrials.gov Identifier: NCT03356392) is an ongoing multicenter, prospective open-label registry of patients undergoing mechanical thrombectomy who have had an acute ischemic stroke caused by a large-vessel occlusion.^14^ Of the 25 participating centers, 11 were excluded from our analysis because of a lack of sufficient 90-day follow-up data. Of the remaining centers, seven hospitals had no privacy policy objections to collecting additional administrative data on their patients. Data extracted from the GSR-ET included baseline characteristics, neurological and neuroradiological findings. Stroke etiology was classified according to the Trial of Org 10,172 in Acute Stroke Treatment (TOAST) classification.^15^ Functional outcome, such as modified Rankin Scale (mRS) at discharge and after 90 days, National Institutes of Stroke Scale (NIHSS) at hospital discharge, were also reported. Favorable functional outcome was defined as a mRS 0–2.

Adverse events were reported and categorized into four time-based groups: (1) periprocedural events, including device malfunction, dissection, clot migration, intracerebral hemorrhage (ICH), vasospasm, stroke, neurological deterioration and other complications, (2) events occurring after 24 h, such as groin hematoma, groin aneurysm, malignant middle cerebral artery infarction, myocardial injury, recurrent stroke, and others, (3) events at discharge, including groin hematoma, groin aneurysm, myocardial injury, recurrent stroke, and other complications, (4) events up to 90 days post-procedure, comprising stroke, ICH, myocardial infarction and other.

Quality of life after 90 days was measured by the EQ5D-3L, calculated as index values. Data extracted on adverse events were collected at the timepoint at intervention, after 24 h, at discharge, and after 90 days. The 90-day follow-ups were either conducted as an onsite visit or via a standardized telephone interview by an experienced stroke neurologist. To assess frailty, we calculated each patient’s Hospital Frailty Risk Score (HFRS) based on the International Classification of Disease (ICD)-10 diagnostic codes at admission. Stroke, as the qualifying event for admission, was excluded in our HFRS analysis. The HFRS, an ICD-10 based score, is used to predict the risk of frailty, and is capable of forecasting adverse health care outcomes and mortality in frail patients. We divided the score into three categories according to the original paper; 0–5 (low-risk), >5–15 (intermediate-risk), and >15 (high risk).^16^

Our study was centrally approved by the Ethics Committee of Ludwig-Maximilians-University LMU, Munich (689-15), as the leading ethics committee. Additional approval was obtained from local ethics committees or institutional review boards according to local regulations. Written informed consent was obtained from patients in centers where it was mandatory. In other centers informed consent was not required in accordance with the institutional review board approval because no study-specific procedures were performed and data sampling from patients undergoing endovascular treatment is already mandatory by national law for quality control reasons.

## Statistical analysis

For descriptive statistics, continuous data are presented using median and interquartile range (IQR), categorial variables are presented as absolute and relative frequencies (%). Pearson’s Chi-squared test for categorial variables and Kruskal-Wallis-Test for continuous variables were used for group comparisons. Binary logistic regression analysis was used to calculate the impact of frailty on functional outcome and mortality after 90 days. We conducted a multivariate logistic regression analysis to calculate the odds ratio with corresponding 95% confidence intervals (CI) to analyze the influence of frailty on functional neurological outcome and mortality after 90 days. The multivariate model was adjusted for common predictors influencing neurological outcome and mortality, that is: Age, Alberta Stroke Programme Early CT-Score (ASPECTS), Thrombolysis in Cerebral Infarction (mTICI) Score, intravenous thrombolysis, baseline NIHSS, periprocedural adverse treatment events, and, pre-stroke mRS. All adjustment parameters were pre-defined before data analysis. We considered our results as statistically significant when *p*-value <0.05. All statistical analyses were performed in SPSS vs 25 (IBM Corp., Armonk, NY, USA).

## Results

**Figure 1.**
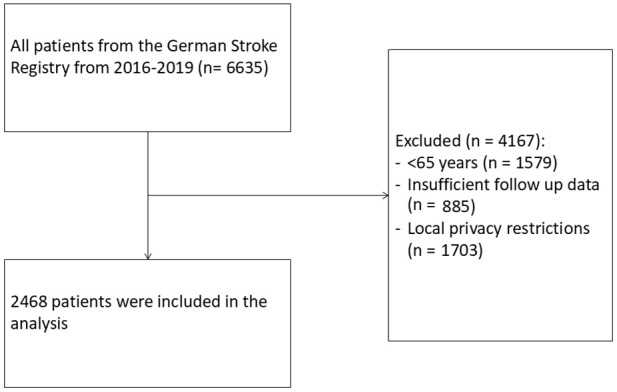
Flowchart of patient selection.

The complete GSR dataset contained 6635 patients. From the 6635 patients, 5056 were ⩾65 years. Of these patients, 885 patients had to be excluded due to sufficient follow up data at 3 months. Furthermore 1703 patients had to be excluded due to local privacy restrictions. A total of 2468 patients were included in the final analysis (Figure 1).

Median age was 79 years (IQR 74–84), mean pre-stroke mRS was 0 (IQR 0–2). Median baseline NIHSS was 15 (IQR 10–19) and 6 (IQR 0–13) at discharge. The median HFRS was 1.1 (IQR 0–2.95) and 449 patients (18.2%) were classified as frail with an HFRS > 5. Among the cohort, 2009 patients (71.7%) had a low frailty risk, 389 (15.8%) had an intermediate frailty risk and 60 patients (2.4%) were at high frailty risk. Distribution of baseline, neurological and neuroradiological characteristics of the aforementioned frailty-risk categories are shown in Table 1. Pre-stroke mRS was significantly higher in frail patients compared to non-frail patients (2 (IQR 1–3) vs 1 (IQR 0–3) vs 0 (IQR 0–1); *p* < 0.001). Additionally frail patients were more likely to live in a nursing home prior to stroke (18 (32.1%) vs 77 (20.9%) vs 136 (7.2%); *p* < 0.001). However, there was no significant difference in baseline NHISS scores between frail and non-frail patients (14 (IQR 19–19)) vs 15 (IQR 11–19) vs 15 (IQR 10–19); *p* = 0.295), even though the mRS was different.

**Table 1.** Baseline characteristics of the various frailty groups.

Baseline characteristics	Low-frailty risk (HFRS < 5) (*n* = 2009)	intermediate-frailty risk HFRS (>5–15) (*n* = 389)	High-frailty risk (HFRS > 15) (*n* = 60)
Sex			
Female, *n* (%)	1076 (53.6)	229 (58.9)	43 (71.4)
Age, years, median (IQR)	79 (74–84)	81 (76–87)	80 (76–85)
Pre-stroke mRS			
0, *n* (%)	1255 (65.5)	145 (39.9)	11 (20)
1, *n* (%)	255 (13.3)	62 (17.1)	10 (18.2)
2, *n* (%)	173 (9)	46 (12.7)	8 (14.5)
Pre-stroke living status			
Home, *n* (%)	1705 (89.9)	264 (71.7)	33 (58.9)
Nursing care at home, *n* (%)	58 (3.1)	27 (7.3)	5 (8.9)
Nursing home, *n* (%)	136 (7.2)	77 (20.9)	18 (32.1)
Comorbidities			
Hypertension, *n* (%)	1632 (82)	334 (87.4)	49 (83.1)
Diabetes, *n* (%)	411 (20.7)	111 (29.1)	15 (25.4)
Dyslipidemia, *n* (%)	819 (41.3)	171 (44.9)	29 (49.2)
Atrial fibrillation, *n* (%)	995 (50.1)	226 (59.3)	27 (45.8)
History of smoking, *n* (%)	265 (15.7)	62 (17.8)	8 (15.4)
Neurological and neuroradiological characteristics			
NIHSS at admission (IQR)	15 (10–19)	15 (11–19)	14 (9–19)
Etiology			
Cardioembolism, *n* (%)	1189 (59.7)	232 (60.7)	31 (52.5)
Large-artery atherosclerosis, *n* (%)	424 (21.3)	77 (20.2)	14 (23.7)
Stroke of other etiology, *n* (%)	69 (3.5)	9 (2.4)	3 (3.9)
Stroke of unknown etiology, *n* (%)	310 (15.6)	64 (16.8)	11 (18.6)
Small-vessel occlusion, *n* (%)	-	-	-
Intravenous thrombolysis, *n* (%)	1049 (52.8)	195 (50.6)	31 (51.7)
Vertebrobasilar stroke *n* (%)	243 (12.5)	53 (14.1)	8 (13.6)
ASPECTS, (IQR)	8 (6–9)	8 (6–9)	9 (7–10)
mTICI			
2b, *n* (%)	727 (37.1)	134 (35.1)	20 (35.1)
3, *n* (%)	900 (46)	186 (48.7)	31 (54.4)
Length of stay, days (IQR)	8 (8)	9 (8)	7 (8)
Door to groin time, min (IQR)	64 (38–102)	67.5 (45–95)	80 (54–106)

HFRS: hospital frailty risk score; *n*: number, IQR: interquartile range; NIHSS: National Institute of Health Stroke Score; mRS: modified Rankin Scale; ASPECTS: Alberta Stroke Program Early CT Scale; mTICI: modified thrombolysis in cerebral infarction scale.

Furthermore, there was no significant difference in the rate of intravenous thrombolysis (*p* = 0.738) or the length of stay in the hospital (*p* = 0.354).

Patients differed significantly with respect to ASPECTS with patients in the high HFRS group having the highest ASPECT score (*p* = 0.016). Besides door-to-groin time (DTN) differed with high frailty-risk patients having the longest DTN times (*p* = 0.020; Table 1).

**Figure 2.**
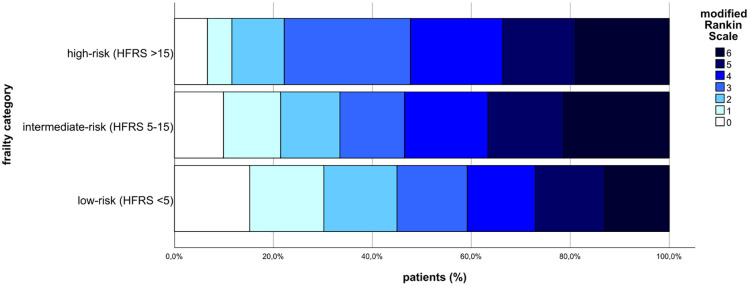
Functional outcome measured by the modified Rankin Scale after 90 days.

A closer examination at functional outcomes revealed a significant difference in NIHSS at discharge (5 (0–13) vs 8 (1–16) vs 6 (0–12); *p* = 0.027). Similarly, the mRS at discharge was significant different (4 (2–5) vs 5 (4–5) vs 4 (2–5); *p* = 0.001). However, after 90 days this observation had disappeared, when mRS was the higher in the high-risk and intermediate-risk frailty groups compared to the low-frailty risk category (5 (4–6) vs 5 (4–6) vs 4 (2–6); *p* < 0.001; Figure 2).

Moreover, quality of life, measured by EQ5D-3L was lower in patients with frailty (0.59 (0.59) vs 0.70 (0.74) vs 0.81 (0.62); *p* = 0.008) after 90 days. Additionally frail patients were less likely to live independently at home compared to non-frail patients (13 (24.1) vs 75 (21.3) vs 623 (36.9); *p* < 0.001; Table 2).

**Table 2.** Functional neurological outcome and living status after 90 days.

Functional outcome	Low-frailty risk (HFRS < 5) (*n* = 2009)	Intermediate-frailty risk (HFRS 5–15) (*n* = 389)	High-frailty risk (HFRS > 15) (*n* = 60)	*p*-Value
NIHSS at discharge	6 (0–12)	8 (1–16)	5 (0–13)	0.027
mRS at discharge	4 (2–5)	5 (4–5)	4 (2–5)	0.001
Outcome at 90 days				
mRS at 90 days	4 (2–6)	5 (4–6)	5 (4–6)	<0.001
Favorable functional outcome (mRS 0–2), *n* (%)	625 (31.7)	78 (20.6)	8 (13.8)	<0.001
Mortality at 90 days, *n* (%)	634 (32.2)	173 (45.6)	24 (41.4)	<0.001
EQ5D-3L at 90 days (*n* = 1040)	0.813 (0.62)	0.701 (0.74)	0.589 (0.59)	0.008
Living status at 90 days				<0.001
Home (%)	623 (36.9)	75 (21.3)	13 (24.1)	
Nursing at home (%)	107 (6.3)	24 (7)	7 (13)	
Nursing home (%)	165 (9.8)	47 (13.7)	5 (9.3)	
Residential rehabilitation (%)	118 (7)	19 (5.5)	3 (5.6)	
Semi-residential rehabilitation (%)	15 (0.9)	2 (0.6)	0 (-)	
Hospital (%)	28 (1.7)	5 (1.5)	2 (3.7)	

HFRS: hospital frailty risk score; *n*: number; IQR: interquartile range; NIHSS: National Institute of Health Stroke Score; mRS: modified Rankin Scale.

Multivariate logistic regression analysis confirmed the significant association between frailty on functional outcome after 3 months after, even adjusting for known predictors for a poor functional outcome (OR: 0.962, 95% CI: 0.929–0.997; *p* = 0.033; Table 3).

**Table 3.** Multivariate logistic regression analysis regarding prediction of favorable neurological outcome (mRS 0–2) and mortality after 90 days.

Favourable neurological outcome	OR	95% CI	*p*-Value
HFRS	0.962	0.929–0.997	0.034
Sex	1.036	0.799–1.344	0.789
Pre-stroke mRS	0.571	0.494–0.660	<0.001
NIHSS at admission	0.886	0.886–0.907	<0.001
Age	0.920	0.902–0.938	<0.001
ASPECTS	1.178	1.088–1.277	<0.001
Intravenous thrombolysis	1.718	1.319–2.237	<0.001
mTICI	1.720	1.498–1.974	<0.001
Adverse treatment events	0.261	0.187–0.363	<0.001
Mortality after 90 days			
HFRS	1.033	1.004–1.063	0.024
Pre-stroke mRS	1.352	1.234–1.481	<0.001
NIHSS at admission	1.082	1.061–1.103	<0.001
Age	1.077	1.059–1.096	<0.001
ASPECTS	0.903	0.848–0.961	0.001
Intravenous thrombolysis	0.657	0.516–0.835	0.001
mTICI	0.654	0.593–0.721	<0.001
Adverse treatment events	3.489	2.687–4.529	<0.001

HFRS: hospital frailty risk score; *n*: number; IQR: interquartile range; NIHSS: National Institute of Health Stroke Score; ASPECTS: Alberta Stroke Program Early CT Scale; mTICI: modified thrombolysis in cerebral infarction scale.

We obtained similar results regarding mortality. Multivariate logistic regression analysis indicated a significant influence of frailty on mortality after 90 days, even after adjusting for known predictors associated with higher mortality after stroke (OR 1.033, 95% CI: 1.004–1.063, *p* = 0.024)

High-risk frailty patients exhibit a significantly lower risk of periprocedural complications (6 (10.3%) vs 77 (20.1%) vs 296 (15%); *p* = 0.022) and adverse events during their hospital stay (20 (35.1) vs 192 (52%) vs 846 (45%); *p* = 0.011; Table 4). However, that changed after 90 days, as high-risk frail patients demonstrated a significantly higher risk of adverse events after 90 days compared to those with an intermediate- or low-frailty risk (9 (30%) vs 24 (13.8%) vs 135 (14%); *p* = 0.048).

**Table 4.** Adverse events in the different frailty-risk groups.

Adverse events	Low risk (HFRS < 5) (*n* = 2009)	Intermediate risk (HFRS 5–15) (*n* = 389)	High risk (HFRS > 15) (*n* = 60)	*p*-Value
Periprocedural complications, *n* (%)	296 (15)	77 (20.1)	6 (10.3)	0.022
Adverse treatment events after 24 h, *n* (%)	515 (26.2)	98 (25.7)	9 (15.3)	0.166
Adverse events at discharge, *n* (%)	846 (45)	192 (52)	20 (35.1)	0.011
Adverse events at 90 days, *n* (%)	135 (14)	24 (13.8)	9 (30)	0.048

HFRS: hospital frailty risk score; *n*: number.

## Discussion

Advanced age is associated with an increased risk of a unfavorable functional outcome following LVOS treated with endovascular treatment.^17^ Increasing age is one major risk factor for a poor neurological outcome.^18^ Understanding the factors that contribute to an unfavorable functional outcome in older patients is crucial in our aging society. Our study is the first large multicentric cohort to identify frailty as measured by pre-stroke HFRS as an indicator of unfavorable neurological outcomes and increased mortality in patients with LVOS. While our study did not clarify the reasons why frailty is linked to a greater likelihood of unfavorable outcomes and mortality after LVOS, we observed that periprocedural adverse events and adverse events during the hospital stay were significantly lower in the high-frailty risk group compared to the intermediate- and low-frailty risk groups. This finding is noteworthy, given that frail patients present more often health-care associated infections^19^ and delirium.^20^ After 90 days, however, a significant increase in adverse events was observed in high-frailty risk patients compared to those with a low or intermediate-frailty risk. This finding suggests that complications in frail patients may occur post-discharge, warranting further investigation into the underlying reasons. An alternative explanation for the lower adverse event rate could be a more conservative treatment approach during endovascular treatment, which may minimize the risk of periprocedural complications. Moreover, high-risk frail patients have higher ASPECTS. The higher ASPECTS indicates a potential selection bias. It is possible, that only frail patients, with high ASPECTS are referred to endovascular treatment. Another notable finding in our study is that door-to-groin-time is longest in high-risk frail patients. This delay may be due to a perception among healthcare providers that treatment times are less critical in high-frailty risk patients. Alternatively, it may be that the families of these patients are more frequently consulted to determine their approval for such a complex treatment like mechanical thrombectomy in their relatives. These findings underscore the need for further research into potential avoidable bias factors in treatment algorithms. NIHSS at admission and discharge as well as and stroke severity are similar across all groups indicating that endovascular treatment is as effective in patients suffering from frailty compared to those without. However, the reasons for worse neurological outcomes in frail patients after 90 days are unclear. One reason for this may be that frail people have less rehabilitation capacity and may therefore be more likely to be transferred to nursing homes rather than rehabilitation facilities.^21^ In contrast, the HERMES data indicate that the primary benefit of endovascular treatment lies in preventing severe disabilities rather than reducing mortality. Mortality rates after 90 days were similar between the control and intervention groups in the HERMES collaborators’ meta-analysis.^22^

Moreover, frail patients experience a lower quality of life after mechanical thrombectomy after 90 days compared to their non-frail counterparts. A phenomenon also observed in a Norwegian study linking frailty to decreased quality of life post-stroke in minor strokes.^23^ Quality of live is apparently not influenced by the event of a stroke alone but by many factors of whom HFRS may collect some.

Although using a large, multi-center data set, our study has several limitations. First, this is a retrospective study and we had to exclude patients from our database due to missing data because of local privacy issues. But since we had to exclude complete hospitals from the study, bias because of missing data is limited. Being a German study, we relied on data collected at each patient’s admission, and there is no central database in Germany for ICD-10 codes of each patient, unlike the Hospital Episode Statistics inpatient database in England, which contains up to 20 ICD-10 codes of all patients admitted to National Health Service hospitals. As a result, our score calculations depended on the recorded patient data at admission, potentially overlooking some pre-existing diagnoses and leading to lower HFRS scores compared with more comprehensive data. In addition, certain ICD-10 codes included in the HFRS, such as unspecified fall (W19) or another fall on the same level (W18), do not exist in the German ICD-10 system.

Furthermore, we cannot exclude selection bias. Extremely frail persons may not present for hospital admission when exhibiting stroke symptoms or may receive rather conservative treatment through intravenous thrombolysis, or no treatment instead of mechanical thrombectomy, particularly if they are in a palliative setting. Thus, our high-risk frailty group numbers may not accurately represent the true incidence of strokes in this patient population. It is also important to note that the HFRS has not yet been validated in a large, general stroke cohort. Since there are various scores for predicting frailty, that is, the Edmonton frail scale^24^ and clinical frailty score,^25^ we selected the HFRS due to its ease of calculation using administrative data, validation in a large cohort,^16^ and widespread use in studies of other acute conditions like transcatheter aortic valve replacement^26^ or myocardial infarction.^27^ Research has been conducted on the relationship between mechanical thrombectomy and frailty in large-vessel occlusion stroke using the HFRS, with small monocentric analyses demonstrating its impact on neurological outcome^12^ and on favorable outcome and mortality after 90 days.^13^ Our study has now replicated and confirmed these findings in a large multicentric cohort.

We believe that our findings are particularly valuable because frailty, especially in its early stages, is potentially reversible through interventions like increased physical activity, improved nutrition, and cognitive interventional approaches.^28^ Our data reinforce the evidence that frailty has a detrimental impact on outcomes, particularly in conditions with high socio-economic and healthcare burdens, such as stroke. The growing evidence on the negative effects of frailty calls for a rethinking of primary prevention strategies, emphasizing a personalized approach to reduce or prevent frailty, ultimately improving functional outcome in stroke patients.

In terms of healthcare economics, our findings suggest that the cost of reducing frailty could be offset by the high direct and indirect costs associated with long-term care of stroke survivors, particularly those affected by LVOS. Incorporating frailty assessments into clinical practice could play a pivotal role in guiding decisions related to hyperacute therapies and setting appropriate rehabilitation goals. By accounting for frailty in the management of older stroke patients, we may be able to reduce the risk of adverse outcomes following mechanical thrombectomy for LVOS, potentially improving both patient recovery, and long-term quality of life. However, assessing frailty in an emergency setting may lead to ethical questions. Since frailty is a predictor for a negative outcome, not only in stroke, assessing frailty in an emergency setting may lead to the risk of leaving vulnerable patients without adequate stroke treatment. Instead, frailty assessments should be used to inform personalized treatment plans for this high-risk patient group.

To address these challenges, we propose developing evidence-based care pathways that account for the unique needs and vulnerabilities of frail patients. This could involve providing additional support and resources for frail patients and their caregivers, as well as developing tailored and patient-centered treatment plans, including rehabilitation strategies. Furthermore, we highlight the need to investigate why high-risk frail patients benefit from endovascular treatment during hospitalization but experience more complications and worse functional outcomes after 90 days. A better understanding of this issue could lead to significant benefits for patients suffering from frailty. Ultimately, improving outcomes for frail patients with stroke requires a nuanced and comprehensive understanding of the complex interplay between frailty, stroke, and other comorbidities.
